# Trends in Cigarette Smoking Rates and Quit Attempts Among Adults With and Without Diagnosed Diabetes, United States, 2001–2010

**DOI:** 10.5888/pcd10.120259

**Published:** 2013-09-19

**Authors:** Amy Z. Fan, Valerie Rock, Xuanping Zhang, Yan Li, Laurie Elam-Evans, Lina Balluz

**Affiliations:** Author Affiliations: Valerie Rock, Xuanping Zhang, Laurie Elam-Evans, Lina Balluz, Centers for Disease Control and Prevention, Atlanta, Georgia; Yan Li, Georgia Department of Community Health, Atlanta, Georgia.

## Abstract

**Introduction:**

Quitting smoking is a critical step toward diabetes control. It is not known whether smoking rates in adults with diabetes are similar to rates among adults who do not have the disease or whether people with diabetes have increased motivation to quit. We examined prevalence trends of current smoking and quit attempts among US adults with and without diagnosed diabetes from 2001 through 2010.

**Methods:**

We used data from the 2001 through 2010 Behavioral Risk Factor Surveillance System, a state-based telephone survey of noninstitutionalized US adults, and conducted linear trend analysis and log linear regression.

**Results:**

The adjusted prevalence of cigarette smoking among adults with diagnosed diabetes was 9% less than adults without diagnosed diabetes (adjusted prevalence ratio [APR], 0.91; 99% confidence interval [CI], 0.89−0.93). Declines in smoking prevalence were greater among adults without diabetes than adults with diagnosed diabetes (*P* < .001). Among smokers, the adjusted prevalence of quit attempts among adults with diagnosed diabetes was 13% higher than among adults without diagnosed diabetes (APR, 1.13; 99% CI, 1.11−1.15). Among adult smokers with diagnosed diabetes, quit attempts were stable over time for those aged 18 to 44 years and those with a high school education or less. Quit attempts were also stable for older smokers, non-Hispanic African Americans, and Hispanic smokers, regardless of diagnosed diabetes status.

**Conclusion:**

A large proportion of smokers with diagnosed diabetes seemed to have quit smoking, but more research is needed to confirm success and how difficult it was to achieve.

## MEDSCAPE CME

Medscape, LLC is pleased to provide online continuing medical education (CME) for this journal article, allowing clinicians the opportunity to earn CME credit.

This activity has been planned and implemented in accordance with the Essential Areas and policies of the Accreditation Council for Continuing Medical Education through the joint sponsorship of Medscape, LLC and Preventing Chronic Disease. Medscape, LLC is accredited by the ACCME to provide continuing medical education for physicians.

Medscape, LLC designates this Journal-based CME activity for a maximum of 1 **AMA PRA Category 1 Credit(s)™**. Physicians should claim only the credit commensurate with the extent of their participation in the activity.

All other clinicians completing this activity will be issued a certificate of participation. To participate in this journal CME activity: (1) review the learning objectives and author disclosures; (2) study the education content; (3) take the post-test with a 70% minimum passing score and complete the evaluation at www.medscape.org/journal/pcd (4) view/print certificate.

**Release date: September 18, 2013; Expiration date: September 18, 2014**

### Learning Objectives

Upon completion of this activity, participants will be able to:

- Compare the prevalence of cigarette smoking among adults with and without diabetes
- Distinguish demographic factors associated with lower rates of cigarette smoking
- Compare rates of smoking cessation attempts among adults with and without diabetes
- Distinguish variables associated with higher rates of smoking cessation attempts among adults with diabetes

**EDITORS**

Caran Wilbanks, Editor, *Preventing Chronic Disease*. Disclosure: Caran Wilbanks has disclosed the following relevant financial relationship: Partner is employed by McKesson Corporation.

**CME AUTHOR**

Charles P. Vega, MD, Associate Professor and Residency Director, Department of Family Medicine, University of California, Irvine. Disclosure: Charles P. Vega, MD, has disclosed no relevant financial relationships.

**AUTHORS AND CREDENTIALS**

Disclosures: Amy Z. Fan, PhD; Valerie Rock, MPH; Xuanping Zhang, PhD; Yan Li, MPH; Laurie Elam-Evans, PhD; Lina Balluz, PhD have disclosed no relevant financial relationships.

Affiliations: Valerie Rock, Xuanping Zhang, Laurie Elam-Evans, Lina Balluz, Centers for Disease Control and Prevention, Atlanta, Georgia; Yan Li, Georgia Department of Community Health, Atlanta, Georgia.

## Introduction

Cigarette smoking among people with diabetes is associated with an elevated risk of cardiovascular disease and stroke, increased insulin resistance, and various complications such as diabetic nephropathy, retinopathy, neuropathy, and lower extremity amputations (1–3). Previous studies have reported that people with diabetes are as likely to smoke as those without the disease (4,5). Trend analyses indicate that the overall age-adjusted current smoking prevalence among people with diabetes remained unchanged from 1990 to 2001, despite significant reductions in cigarette smoking in the general population during the same time period (4,6). From 1990 to 2001, significant decreases in smoking prevalence among people with diabetes were observed among African Americans and those aged 65 years or older, 2 populations that are disproportionately burdened by the disease and its related complications (4).

Because of the increased risk for comorbidity and mortality among people with diabetes, smoking cessation — not just a reduction in cigarette consumption — is recommended (7). Although there is limited research on the efficacy of smoking cessation interventions for smokers with diabetes, the US Public Health Service and the American Diabetes Association suggest that integration of tobacco use dependence interventions with diabetes management and educational programs may be an effective strategy to increase quit attempts and reduce smoking prevalence among people with diabetes (7,8).

This study seeks to update and build on the existing literature related to cigarette smoking among people with diabetes. We used data from the Behavioral Risk Factor Surveillance System (BRFSS) for 2001 through 2010 to examine the trend in prevalence of current cigarette smoking and quit attempts among the US adults aged 18 years or older by diagnosed diabetes status.

## Methods

BRFSS is an ongoing, state-based, telephone health survey conducted by the random-digit dialing of noninstitutionalized US adults (9,10). BRFSS collects data on sociodemographics, chronic illness, health behaviors, access to health care, and other health-related information on an annual basis. BRFSS can produce national estimates because of its large sample size. For each year, we used poststratification weights for all estimates to account for the respondent’s probability of being selected and the age-, race-, and sex-specific populations from the US Census for the state or area. We used these weights to calculate the state smoking prevalence estimates; 95% confidence intervals (CIs) also were calculated. BRFSS methods, including the weighting procedure, are described elsewhere (9). All BRFSS questionnaires, data, and reports are available on the official BRFSS website (www.cdc.gov/brfss). The state-specific cooperation rates, CASRO (Council of American Survey Research Organizations) response rates, and other outcome rates by state are available in annual data quality reports (2,11). A CASRO response rate is an outcome rate with the number of complete and partial interviews in the numerator and an estimate of the number of eligible units in the sample in the denominator. The median CASRO response rates from 2001 through 2010 ranged from 50.6% to 58.3%.

### Measures

Diabetes and smoking questions were asked in the core questionnaires of survey years 2001 through 2010. The core questions were administered to all respondents aged 18 years or older.

**Diabetes status.** Respondents were asked, “Have you ever been told by a doctor that you have diabetes?” If the response was “yes” and the respondent was female, then she was asked, “Was this only when you were pregnant?” Since 2004, another option was added to the response set, “No, prediabetes or borderline diabetes.” To maintain consistency, we combined those respondents who said that they did not have diabetes with those who indicated that they had been told only during a pregnancy and who indicated that they have prediabetes or borderline diabetes. This definition, of necessity, combined people with undiagnosed diabetes and people without diabetes. As the most recent National Diabetes Fact Sheet indicated, 27% (7.0 million of 25.8 million) of the people with diabetes have not had their condition diagnosed (12).

**Current smoking status.** In 2001 through 2010, respondents were asked, “Have you smoked at least 100 cigarettes in your life?” and “Do you now smoke cigarettes every day, some days, or not at all?” Respondents who smoked at least 100 cigarettes and reported smoking every day or on some days now were considered current smokers.

**Quit attempt during the past 12 months.** Current smokers were asked, “During the past 12 months, have you stopped smoking for one day or longer because you were trying to quit smoking?”

### Statistical analysis

We performed the analysis using SAS-callable SUDAAN 11.0.0 (Research Triangle Institute, Research Triangle Park, North Carolina) to account for the complex sampling design of BRFSS. Data were weighted to the respondent’s probability of selection, nonresponse, and landline telephone coverage. Prevalence estimates of current smoking and quit attempts and their respective standard errors by diabetes status were obtained. The temporal linear trend on 2 outcome variables was estimated by the REGRESS procedure with year as the independent variable. We assessed whether the linear trend (slope) differed by diabetes status by including an interaction term of diagnosed diabetes status and year in the regression models. Log linear regression models were applied to obtain adjusted prevalence ratios (APRs) and 99% CIs for cigarette smoking among US adults and quit attempts among smoking adults by diabetes status after adjustment of demographic characteristics. Given the large sample size of BRFSS, we set significance level at *P* < .01 to minimize occurrence of the type I error and obtain robust results.

We also obtained predictive marginals for cigarette smoking and quit attempts by diagnosed diabetes status and year from the log linear regression analysis with adjustment for sex, age, race/ethnicity, education attainment, and marital status.

## Results

The prevalence of cigarette smoking declined from 2001 through 2010 for adults with and without diagnosed diabetes (Table 1). The decline was more remarkable (*P* < .001) among those without diagnosed diabetes (0.67% average annual decline) than among adults with diagnosed diabetes (0.25% average annual decline). A separate analysis showed that the proportion of former smokers is higher (*P* < .001) among adults with diagnosed diabetes (36.3%) than their counterparts without diagnosed diabetes (23.3%), and the proportions were stable during the 10 years (Table 2). The following demographic subgroups with diagnosed diabetes did not demonstrate significant declines of smoking rate: those aged 65 years or older; minority groups; those who had less than a high school education; and those who were previously married (separated, divorced, or widowed).

**Table 1:** Prevalence of Current Cigarette Smoking Among Adults Aged ≥18 Years by Selected Sociodemographic Characteristics and Self-Reported Diabetes Status, United States, Behavioral Risk Factor Surveillance System, 2001–2010^a^

Characteristic	2001 (n = 212,262), % (SE)	2003 (n = 264,375), % (SE)	2005 (n = 355,758), % (SE)	2007 (n = 430,476), % (SE)	2008 (n = 414,070), % (SE)	2009 (n = 432,182), % (SE)	2010 (n = 450,907), % (SE)	Slope for Annual Decline (SE)	*P* Value^b^
**Total**	22.7 (0.2)	22.2 (0.2)	20.5 (0.1)	19.4 (0.1)	18.4 (0.1)	17.9 (0.1)	17.1 (0.1)	−0.65 (0.06)	<.0001
Diagnosed Diabetes	16.8 (0.5)	17.7 (0.5)	16.5 (0.4)	15.5 (0.3)	15.6 (0.4)	15.3 (0.4)	15.4 (0.3)	−0.25 (0.06)	<.0001
No diagnosed diabetes	23.2 (0.2)	22.5 (0.2)	20.8 (0.2)	19.7 (0.2)	18.7 (0.1)	18.2 (0.1)	17.3 (0.1)	−0.67 (0.06)	<.0001
**Age, y**									
**18–44**									.27^c^
Diagnosed Diabetes	27.9 (1.5)	29.7 (1.7)	27.9 (1.6)	24.4 (1.2)	25.8 (1.4)	25.3 (1.6)	24.5 (1.3)	−0.67 (0.17)	.0001
No diabetes	27.2 (0.2)	26.1 (0.3)	23.9 (0.2)	22.3 (0.2)	21.2 (0.2)	20.8 (0.3)	19.5 (0.2)	−0.85 (0.03)	<.0001
**45–64**									.0003^c^
Diabetes	21.1 (0.9)	21.4 (0.8)	20.1 (0.6)	19.2 (0.5)	19.1 (0.5)	18.6 (0.5)	18.8 (0.5)	−0.27 (0.07)	.0003
No diabetes	22.8 (0.3)	22.7 (0.3)	21.1 (0.2)	20.3 (0.2)	19.3 (0.2)	18.6 (0.2)	18.1 (0.2)	−0.55(0.03)	<.0001
**≥65**									.0002^c^
Diabetes	7.6 (0.5)	7.7 (0.5)	7.5 (0.4)	7.6 (0.4)	7.3 (0.3)	7.3 (0.3)	7.7 (0.3)	−0.06 (0.05)	.20
No diabetes	10.5 (0.3)	9.7 (0.3)	9.3 (0.2)	9.3 (0.2)	8.6 (0.2)	8.6 (0.2)	8.5 (0.1)	−0.24 (0.02)	<.0001
**Sex**									
**Men**									<.0001^c^
Diabetes	18.1 (0.8)	18.0 (0.8)	17.6 (0.7)	16.6 (0.6)	16.8 (0.6)	15.6 (0.6)	15.9 (0.5)	−0.31 (0.07)	<.0001
No diabetes	25.5 (0.3)	25.5 (0.3)	23.1 (0.3)	22.0 (0.3)	21.1 (0.2)	20.3 (0.3)	19.4 (0.2)	−0.73 (0.03)	<.0001
**Women**									<.0001^c^
Diabetes	15.6 (0.7)	17.5 (0.7)	15.4 (0.5)	14.4 (0.4)	14.4 (0.4)	15.0 (0.4)	14.8 (0.4)	−0.21 (0.06)	.0003
No diabetes	21.0 (0.2)	19.7 (0.2)	18.6 (0.2)	17.6 (0.2)	16.4 (0.2)	16.2 (0.2)	15.2 (0.1)	−0.62 (0.02)	<.0001
**Race/ethnicity**									
**White**									<.0001^c^
Diabetes	16.5 (0.6)	17.4 (0.6)	16.4 (0.4)	15.3 (0.3)	15.5 (0.4)	15.4 (0.4)	15.0 (0.3)	−0.22 (0.05)	<.0001
No diabetes	23.7 (0.2)	23.0 (0.2)	21.3 (0.2)	20.1 (0.2)	19.0 (0.2)	18.5 (0.2)	17.6 (0.1)	−0.71 (0.02)	<.0001
**African American**									.42^c^
Diabetes	18.3 (1.5)	22.1 (1.6)	18.6 (1.1)	17.6 (1.0)	17.1 (1.0)	17.4 (1.0)	18.5 (0.9)	−0.26 (0.14)	.056
No diabetes	23.2 (0.6)	24.0 (0.6)	21.2 (0.5)	22.1 (0.6)	21.1 (0.5)	20.3 (0.6)	19.3 (0.4)	−0.38 (0.06)	<.0001
**Hispanic**									.10^c^
Diabetes	14.3 (1.6)	12.8 (1.6)	14.1 (1.7)	14.0 (1.3)	14.2 (1.3)	12.4 (1.3)	12.7 (1.0)	−0.26(0.14)	.10
No diabetes	19.4 (0.6)	18.4 (0.6)	17.8 (0.6)	16.5 (0.5)	15.7 (0.5)	14.8 (0.5)	14.2 (0.5)	−0.38 (0.06)	<.0001
**Other^d^**									.25^c^
Diabetes	23.1 (2.6)	21.9 (2.7)	19.2 (1.9)	17.1 (1.5)	17.1 (1.5)	17.2 (1.5)	18.7 (1.5)	−0.55(0.23)	.017
No diabetes	24.9 (0.8)	24.5 (0.8)	21.6 (0.7)	19.7 (0.7)	18.6 (0.6)	19.7 (0.7)	16.8 (0.5)	−0.83 (0.08)	<.0001
**Education**									
**Less than high school diploma**									.025^c^
Diabetes	18.9 (1.2)	19.3 (1.1)	18.5 (1.0)	19.5 (1.0)	18.5 (0.9)	18.0 (0.9)	18.6 (0.8)	−0.08 (0.12)	.49
No diabetes	31.9 (0.6)	31.4 (0.6)	29.7 (0.6)	30.5 (0.6)	29.3 (0.6)	29.5 (0.6)	28.3 (0.6)	−0.38 (0.07)	<.0001
**High school diploma**									<.0001^c^
Diabetes	18.7 (0.9)	19.1 (0.9)	17.2 (0.7)	16.9 (0.6)	17.4 (0.7)	17.7 (0.6)	17.7 (0.6)	−0.17 (0.08)	.04
No diabetes	28.9 (0.3)	29.2 (0.3)	27.2 (0.3)	26.4 (0.3)	25.0 (0.3)	24.9 (0.3)	24.5 (0.3)	−0.56 (0.03)	<.0001
**Some college or more**									<.0001^c^
Diabetes	14.4 (0.7)	16.1 (0.8)	15.2 (0.6)	13.1 (0.4)	13.2 (0.5)	12.9 (0.5)	12.8 (0.4)	−0.31 (0.06)	<.0001
No diabetes	18.2 (0.2)	17.3 (0.2)	15.7 (0.2)	14.7 (0.2)	13.8 (0.2)	13.4 (0.2)	12.5 (0.1)	−0.63 (0.02)	<.0001
**Marital status**									
**Married**									<.0001^c^
Diabetes	14.5 (0.7)	15.3 (0.7)	13.8 (0.5)	12.9 (0.4)	12.6 (0.4)	12.5 (0.4)	13.0 (0.4)	−0.27 (0.06)	<.0001
No diabetes	18.9 (0.2)	18.0 (0.2)	16.6 (0.2)	15.6 (0.2)	14.5 (0.2)	14.0 (0.2)	13.1 (0.1)	−0.64 (0.02)	<.0001
**Previously married^e^**									<.0001^c^
Diabetes	19.0 (0.9)	20.1 (0.9)	18.4 (0.7)	18.6 (0.6)	18.3 (0.7)	17.5 (0.7)	17.8 (0.5)	−0.19 (0.08)	.016
No diabetes	28.7 (0.4)	28.2 (0.4)	26.5 (0.3)	26.4 (0.3)	25.3 (0.3)	24.4 (0.3)	24.9 (0.3)	−0.53 (0.04)	<.0001
**Never married^f^**									.24^c^
Diabetes	25.1 (1.9)	25.5 (1.9)	26.6 (1.9)	21.7 (1.4)	24.2 (1.5)	24.4 (1.5)	21.5 (1.1)	−0.48 (0.18)	.009
No diabetes	29.6 (0.4)	29.5 (0.4)	27.3 (0.4)	25.8 (0.4)	24.6 (0.4)	24.7 (0.4)	23.2 (0.4)	−0.70 (0.04)	<.0001

Abbreviation: SE, standard error.

a The data for years 2002, 2004, and 2006 are not presented because of space limitations.

b The linear trend (slope) test was obtained by regressing the outcome on survey year.

c The *P* value indicates the difference between adults with diagnosed diabetes and those without.

d Other consists of Asian, non-Hispanic; Native Hawaiian/Pacific Islander, non-Hispanic; American Indian/Alaska Native, non-Hispanic; other race, non-Hispanic; and multirace, non-Hispanic.

e Previously married includes those divorced, widowed, or separated.

f Never married includes those never married or members of unmarried couples.

**Table 2:** Percentage of Former Smokers Among Adults With Diagnosed Diabetes and Those Without Diagnosed Diabetes, United States, Behavioral Risk Factor Surveillance System, 2001–2010

Year	Adults With Diagnosed Diabetes, % (SE)	Adults Without Diagnosed Diabetes, % (SE)
2001	37.1 (0.7)	23.6 (0.2)
2002	36.2 (0.7)	23.3 (0.2)
2003	36.5 (0.6)	23.7 (0.2)
2004	35.0 (0.6)	22.4 (0.2)
2005	36.1 (0.6)	23.4 (0.1)
2006	38.4 (0.6)	23.1 (0.2)
2007	35.4 (0.4)	23.1 (0.1)
2008	36.2 (0.4)	23.2 (0.1)
2009	36.2 (0.4)	23.7 (0.1)
2010	36.3 (0.4)	23.8 (0.1)
Total	36.3 (0.2)	23.3 (0.1)

Abbreviation: SE, standard error.

Overall, the estimated prevalence of adult smokers who reported quit attempts for 1 or more days increased an average of 0.5% per year from 2001 to 2010 (55.5% in 2001; 58.8% in 2010) (Table 3). The increase is similar for adults with and without diagnosed diabetes (*P* = .51). Quit attempts among non-Hispanic white smokers with and without diabetes increased during the 10 years. Quit attempts among young adult smokers (aged 18–44 years) with diagnosed diabetes and older adult smokers with or without diabetes (aged ≥65 years) did not change significantly during the 10 years. There were no significant changes in quit attempts during the 10 years among African American and Hispanic smokers regardless of diabetes status. Additionally, no significant changes were seen in quit attempts among smokers with diagnosed diabetes who had a high school education or less. There were no significant changes in quit attempts among smokers with diagnosed diabetes who were married or previously married. In contrast, among smokers with diabetes who had never married, quit attempts increased an average of 1.1% per year (*P* = .005).

**Table 3:** Percentage of Current Adult Smokers With Quit Attempts During the Past 12 Months, by Selected Sociodemographic Characteristics and Self-Reported Diabetes Status, United States, Behavioral Risk Factor Surveillance System, 2001–2010^a^

Characteristic	2001, % (SE)	2003, % (SE)	2005, % (SE)	2007, % (SE)	2008, % (SE)	2009, % (SE)	2010, % (SE)	Slope for Annual Decline (SE)	*P* Value^b^
**Total**	55.5 (0.4)	53.6 (0.4)	55.8 (0.4)	57.6 (0.4)	58.2 (0.4)	59.6 (0.4)	58.8 (0.4)	0.49 (0.04)	<.0001
Diabetes	59.2 (1.6)	57.4 (1.6)	62.3 (1.3)	59.7 (1.2)	62.8 (1.2)	62.3 (1.3)	65.3 (1.0)	0.57 (0.15)	.0001
No diabetes	55.3 (0.4)	53.4 (0.4)	55.3 (0.4)	57.4 (0.4)	57.8 (0.4)	59.4 (0.4)	58.2 (0.4)	0.47 (0.05)	<.0001
**Age, y**									
**18–44**									.62^c^
Diabetes	63.7 (2.9)	64.7 (3.0)	69.9 (2.8)	62.1 (2.7)	67.9 (3.1)	67.5 (3.5)	70.7 (2.6)	0.32 (0.32)	.33
No diabetes	58.6 (0.5)	57.5 (0.5)	59.0 (0.6)	60.9 (0.6)	61.6 (0.6)	62.8 (0.6)	62.1 (0.7)	0.48 (0.06)	<.0001
**45–64**									.34^c^
Diabetes	58.4 (2.2)	53.8 (2.1)	60.2 (1.7)	60.0 (1.5)	62.7 (1.5)	62.5 (1.4)	65.8 (1.2)	0.93 (0.20)	<.0001
No diabetes	49.5 (0.7)	46.7 (0.7)	50.2 (0.6)	53.2 (0.6)	53.2 (0.6)	55.4 (0.5)	53.7 (0.5)	0.74 (0.07)	<.0001
**≥65**									.96^c^
Diabetes	55.0 (3.6)	56.3 (3.3)	57.3 (2.5)	56.1 (2.4)	55.8 (2.3)	54.2 (2.0)	57.4 (1.8)	0.04 (0.30)	.9
No diabetes	50.0 (1.3)	45.4 (1.4)	46.0 (1.2)	48.0 (1.0)	46.8 (1.0)	49.0 (1.0)	49.6 (0.9)	0.02 (0.13)	.85
**Sex**									
**Men**									.36^c^
Diabetes	57.2 (2.4)	56.5 (2.3)	61.3 (2.0)	57.6 (1.8)	59.3 (2.0)	61.0 (1.9)	64.0 (1.6)	0.67 (0.23)	.0029
No diabetes	55.1 (0.6)	52.4 (0.6)	55.1 (0.6)	56.8 (0.6)	56.7 (0.6)	58.9 (0.7)	66.8 (1.2)	0.46 (0.07)	<.0001
**Women**									.98^c^
Diabetes	61.3 (2.1)	58.2 (2.0)	63.4 (1.7)	62.1 (1.4)	67.0 (1.3)	63.8 (1.7)	57.2 (0.6)	0.48 (0.19)	.013
No diabetes	55.6 (0.5)	54.6 (0.5)	55.6 (0.5)	58.2 (0.5)	59.1 (0.5)	59.9 (0.5)	59.3 (0.5)	0.48 (0.06)	<.0001
**Race/ethnicity**									
**White**									.01^c^
Diabetes	53.4 (1.9)	50.5 (1.8)	57.3 (1.5)	55.9 (1.2)	59.2 (1.3)	58.7 (1.4)	61.6 (1.2)	0.87 (0.17)	<.0001
No diabetes	53.2 (0.4)	50.1 (0.4)	52.2 (0.4)	54.2 (0.4)	55.2 (0.4)	56.5 (0.5)	55.4 (0.4)	0.43 (0.05)	<.0001
**African American**									.39^c^
Diabetes	69.2 (4.0)	71.8 (3.6)	74.8 (2.8)	69.0 (2.9)	75.3 (3.0)	73.4 (2.6)	71.2 (2.4)	0.018 (0.34)	.96
No diabetes	64.8 (1.3)	64.3 (1.3)	63.1 (1.3)	66.7 (1.4)	66.0 (1.3)	67.8 (1.4)	69.1 (1.2)	0.34 (0.14)	.018
**Hispanic**									.59^c^
Diabetes	76.0 (4.6)	66.3 (6.4)	72.7 (4.7)	67.7 (4.6)	61.4 (4.9)	63.5 (5.5)	74.0 (3.5)	−0.18 (0.55)	.75
No diabetes	62.1 (1.6)	61.5 (1.7)	64.6 (1.7)	65.9 (1.6)	63.5 (1.7)	65.4 (1.7)	61.8 (1.9)	0.14 (0.19)	.46
**Other^d^**									.38^c^
Diabetes	60.5 (6.1)	67.1 (5.4)	59.2 (5.2)	56.8 (5.0)	67.0 (5.2)	64.0 (4.5)	66.2 (3.9)	0.30 (0.59)	.6
No diabetes	55.0 (1.9)	59.0 (1.7)	60.4 (1.8)	61.9 (1.9)	61.2 (1.8)	64.0 (2.0)	64.6 (1.5)	0.88 (0.20)	<.0001
**Education**									
**Less than high school diploma**									.66^c^
Diabetes	63.2 (3.2)	56.9 (3.1)	61.8 (3.0)	63.2 (2.8)	68.9 (2.5)	62.9 (2.6)	66.5 (2.3)	0.49 (0.31)	.11
No diabetes	57.5 (1.1)	55.2 (1.1)	56.1 (1.1)	58.8 (1.2)	60.1 (1.2)	60.2 (1.2)	57.8 (1.3)	0.34 (0.13)	.006
**High school diploma**									.79^c^
Diabetes	58.2 (2.6)	53.8 (2.6)	62.0 (2.0)	59.2 (1.7)	60.3 (2.1)	61.6 (2.0)	64.6 (1.7)	0.58 (0.24)	.017
No diabetes	53.9 (0.6)	51.6 (0.7)	54.5 (0.7)	56.3 (0.6)	56.7 (0.7)	57.6 (0.7)	57.9 (0.7)	0.51 (0.07)	<.0001
**Some college or more**									.48^c^
Diabetes	57.5 (2.7)	60.7 (2.5)	62.5 (2.1)	58.8 (1.8)	62.2 (1.8)	62.7 (2.1)	65.4 (1.5)	0.65 (0.24)	.0059
No diabetes	55.7 (0.6)	54.3 (0.6)	55.8 (0.6)	57.8 (0.6)	57.9 (0.6)	60.6 (0.6)	58.6 (0.6)	0.48 (0.06)	<.0001
**Marital status**									
**Married**									.93^c^
Diabetes	59.7 (2.3)	56.0 (2.3)	64.4 (1.8)	60.1 (1.8)	60.7 (1.8)	59.8 (1.9)	66.5 (1.5)	0.55 (0.22)	.013
No diabetes	54.5 (0.6)	54.3 (0.6)	55.8 (0.6)	57.8 (0.6)	57.9 (0.6)	60.6 (0.6)	57.3 (0.6)	0.57 (0.06)	<.0001
**Previously married^e^**									.25^c^
Diabetes	61.0 (2.5)	60.3 (2.3)	57.7 (2.0)	60.0 (1.6)	60.7 (2.1)	62.4 (1.9)	63.6 (1.5)	0.31 (0.23)	.17
No diabetes	51.1 (0.8)	50.9 (0.8)	51.9 (0.7)	54.0 (0.7)	55.1 (0.7)	56.1 (0.7)	56.7 (0.7)	0.58 (0.08)	<.0001
**Never married^f^**									.02^c^
Diabetes	52.3 (4.3)	55.5 (4.0)	64.9 (3.5)	58.1 (3.5)	72.0 (2.8)	69.3 (2.9)	64.9 (2.8)	1.13 (0.40)	.0046
No diabetes	59.9 (0.8)	58.2 (0.8)	59.3 (0.9)	60.3 (0.9)	60.7 (0.9)	61.9 (0.9)	60.7 (0.9)	0.19 (0.09)	.045

Abbreviation: SE, standard error.

a The data for 2002, 2004, and 2006 were not presented because of space limitation.

b The linear trend (slope) test was obtained by regressing the outcome on survey year.

c The *P* value indicates the difference between adults with diagnosed diabetes and those without.

d Other consists of Asian, non-Hispanic; Native Hawaiian/Pacific Islander, non-Hispanic; American Indian/Alaska Native, non-Hispanic; other race, non-Hispanic; and multirace, non-Hispanic.

e Previously married includes those divorced, widowed, or separated.

f Never married includes those never married or members of unmarried couples.

Log linear regression analysis showed that the adjusted prevalence of cigarette smoking decreased during the past decade and was 9% lower among adults with diabetes than those without diabetes (APR, 0.91; 99% CI, 0.89−0.93) (Table 4). Quit attempts among smokers increased during the past decade. The adjusted prevalence of a quit attempt among adults with diagnosed diabetes was 13% higher than that for adults without diagnosed diabetes (APR, 1.13; 99% CI, 1.11−1.15).

**Table 4:** Adjusted Prevalence Ratios (APRs) For Cigarette Smoking Among US Adults and Quit Attempts Among Smoking Adults,^a^ United States, Behavioral Risk Factor Surveillance System, 2001–2010

Status or Year	Cigarette Smoking, APR (99% CI)	Quit Attempt Among Smokers, APR (99% CI)
**Diabetes status**		
No diabetes	Reference	Reference
Diabetes	0.91 (0.89−0.93)	1.13 (1.11−1.15)
**Year**		
2001	Reference	Reference
2002	0.99 (0.96−1.01)	1.02 (0.99−1.04)
2003	0.98 (0.96−1.01)	0.96 (0.94−0.99)
2004	0.93 (0.90−0.95)	0.99 (0.96−1.01)
2005	0.92 (0.90−0.94)	1.00 (0.98−1.03)
2006	0.89 (0.86−0.91)	1.03 (1.00−1.05)
2007	0.89 (0.86−0.91)	1.04 (1.01−1.07)
2008	0.84 (0.82−0.87)	1.04 (1.02−1.07)
2009	0.84 (0.81−0.86)	1.06 (1.04−1.09)
2010	0.80 (0.78−0.82)	1.06 (1.03−1.09)

Abbreviation: CI, confidence interval.

a APRs and CIs were obtained from log-linear regression models after adjustment for sex, age, race/ethnicity, education attainment, and marital status.

Although cigarette smoking and quit attempts fluctuated during the past decade, the prevalence of cigarette smoking decreased and the prevalence of quit attempts increased over time for adults with and without diagnosed diabetes (Table 5). Up to 2010, the adjusted prevalence of cigarette smoking was essentially no different among adults with diagnosed diabetes than among those without diagnosed diabetes. The prevalence of quit attempts was higher among adults with diagnosed diabetes than those without diagnosed diabetes.

**Table 5:** Predicted Margins of Cigarette Smoking Among US Adults and Quit Attempts Among Smoking Adults by Diabetes Status^a^, United States, Behavioral Risk Factor Surveillance System, 2001–2010

Smoking Status/Year	With Diagnosed Diabetes, % (SE)	Without Diagnosed Diabetes, % (SE)
**Cigarette smoking**		
2001	18.6 (0.6)	22.4 (0.2)
2002	19.4 (0.6)	22.1 (0.2)
2003	19.6 (0.6)	22.0 (0.2)
2004	18.6 (0.5)	20.5 (0.1)
2005	18.6 (0.5)	20.5 (0.1)
2006	17.4 (0.5)	19.8 (0.2)
2007	17.8 (0.4)	19.8 (0.2)
2008	17.9 (0.4)	18.8 (0.1)
2009	17.7 (0.4)	18.6 (0.1)
2010	17.9 (0.4)	17.8 (0.1)
**Quit attempts among smokers**		
2001	61.9 (1.7)	55.3 (0.4)
2002	64.0 (1.5)	56.2 (0.4)
2003	59.3 (1.6)	53.2 (0.4)
2004	59.9 (1.6)	54.7 (0.4)
2005	64.9 (1.3)	55.2 (0.4)
2006	64.6 (1.6)	56.7 (0.4)
2007	62.8 (1.2)	57.4 (0.4)
2008	65.5 (1.3)	57.7 (0.4)
2009	64.7 (1.3)	59.0 (0.4)
2010	68.0 (1.1)	58.5 (0.4)

Abbreviation: SE, standard error.

a Predictive marginals were obtained from log-linear regression models after adjustment for sex, age, race/ethnicity, education attainment, and marital status.

## Discussion

Our study indicated that the prevalence of cigarette smoking declined from 2001 through 2010 for adults with and without diagnosed diabetes. However, the decline in smoking prevalence was more remarkable among adults without diagnosed diabetes than adults with diagnosed diabetes. Overall, the prevalence of quit attempts among smokers increased from 2001 through 2010, although there were fluctuations throughout the time period. The adjusted prevalence of quit attempts among adult smokers with diagnosed diabetes was 13% higher than among adult smokers without diagnosed diabetes. These findings should be interpreted in light of the fact that the smoking rate among adults with diagnosed diabetes was already below that of those without diagnosed diabetes and the proportion of former smokers was always higher among adults with diagnosed diabetes than those without.

Consistent with the findings from previous studies (4,13), our findings showed a higher prevalence of current smoking among young adults, men, adults with lower education attainment, and adults who had never married or previously married (13). However, a Ford et al (4) study showed the age-standardized prevalence of cigarette smoking was similar in adults with and without diagnosed diabetes and remained stable from 1990 through 2001. The study also reported a significant decrease in smoking prevalence among people with diabetes among African Americans and those aged 65 years or older. Findings from our study revealed a different picture. The discrepancy might be partially explained by different statistical approaches used in these 2 studies. We did not use age-standardized prevalence estimates in the trend analysis because this study is designed to examine the change in the actual burden of cigarette smoking during the last decade. Nonetheless, age is closely related to prevalence of diagnosed diabetes, cigarette smoking, and smoking cessation. Log-linear regression models with demographic covariates adjustment (including age) revealed discrepant smoking rates based on diagnosed diabetes status. However, smoking rates declined from 2001 through 2010 in different slopes for adults with and without diabetes. In 2010, the adjusted smoking rates were no longer significantly different.

Our findings demonstrate a complicated picture about differential smoking behavior by diabetes status in the United States during 2001 through 2010. The prevalence of cigarette smoking among adults with diabetes was lower than that among adults without diagnosed diabetes. Furthermore, quit attempts among adults with diabetes were higher than for adults without diagnosed diabetes, but the prevalence of smoking declined more remarkably among adults without diabetes from 2001 through 2010. Floor effects can be an explanation, although other factors may have contributed to this trend. Research has shown that significant health events and the risk of adverse health outcomes may influence behavior change, including smoking cessation (14,15). The diagnosis of a chronic disease may increase the odds of smoking cessation (16–19) and produce a greater desire for smoking cessation (20). However, research also suggests that smokers with diabetes are less active in their diabetes self-care and less compliant with recommendations for diabetes management (21). Additionally, they may be more likely to report feelings of depression than nonsmokers with the disease and have a low readiness to quit smoking (21,22).

A growing body of literature describing the bidirectional nature of smoking and diabetes further complicates the interpretation and translation of these results. Although a diagnosis of diabetes may prompt a quit attempt or successful cessation, smoking cigarettes may actually lead to a diabetes diagnosis (23). This may contribute to the slower decline in smoking prevalence among smokers with diabetes. Despite the risks associated with smoking and diabetes, few intervention studies address smoking prevention and cessation in the population with diabetes (24–27).

There were several limitations in this study. First, there was noncoverage bias in BRFSS. Only landline residential telephone households were sampled in the telephone survey. Institutionalized populations (eg, prisons, school dormitories, nursing homes, military bases) and cellular-telephone–only households were not included in the sample. One study (28) indicated that relative bias for the estimate of current smoking from a landline BRFSS survey in 2008 was about 10%. In other words, current smoking was largely underestimated by excluding cellular-telephone–only households. Second, as is the case in other telephone surveys, the overall response rates in BRFSS are declining over the years. Third, there might be social desirability bias. With smoking behaviors becoming less popular in the population, smokers may not admit their status in a telephone survey. Fourth, BRFSS uses self-report to determine diabetes status. It does not allow us to distinguish those with undiagnosed diabetes from those without diabetes. We could not differentiate type 1 from type 2 diabetes with this data set. This self-report approach may also introduce misclassification bias, but the overall influence of misclassification is unknown. In addition, BRFSS uses a multistage sampling design primarily to generate state or area estimates. The median prevalence among all states and the District of Columbia is generally comparable to overall national estimates from other surveys (29).

This study delineated the cigarette smoking prevalence among demographic subgroups of US populations and their secular trends from 2001 through 2010 by diabetes status using a large state-based survey. Although prevalence of cigarette smoking has generally declined during the last decade, our study found that rates declined less among young adults with diagnosed diabetes and among minority groups and those with lower levels of education. Given the enhanced risk of comorbidity and premature mortality from the combination of smoking and diabetes, additional research is needed to examine the effectiveness of integrating recommended clinical interventions for smokers (eg, counseling, cessation medications) (30) with diabetes self-management and education programs, especially to those who are newly diagnosed, and to assess the effect of targeted cessation interventions for this population with chronic disease.

## Figures and Tables

**Table:** 

**1. The activity supported the learning objectives.**				
**Strongly Disagree**				**Strongly Agree**
1	2	3	4	5
**2. The material was organized clearly for learning to occur.**				
**Strongly Disagree**				**Strongly Agree**
1	2	3	4	5
**3. The content learned from this activity will impact my practice.**				
**Strongly Disagree**				**Strongly Agree**
1	2	3	4	5
**4. The activity was presented objectively and free of commercial bias.**				
**Strongly Disagree**				**Strongly Agree**
1	2	3	4	5

## Post-Test Information

To obtain credit, you should first read the journal article. After reading the article, you should be able to answer the following, related, multiple-choice questions. To complete the questions (with a minimum 70% passing score) and earn continuing medical education (CME) credit, please go to http://www.medscape.org/journal/pcd. Credit cannot be obtained for tests completed on paper, although you may use the worksheet below to keep a record of your answers. You must be a registered user on Medscape.org. If you are not registered on Medscape.org, please click on the "Register" link on the right hand side of the website to register. Only one answer is correct for each question. Once you successfully answer all post-test questions you will be able to view and/or print your certificate. For questions regarding the content of this activity, contact the accredited provider, CME@medscape.net. For technical assistance, contact CME@webmd.net. American Medical Association's Physician's Recognition Award (AMA PRA) credits are accepted in the US as evidence of participation in CME activities. For further information on this award, please refer to http://www.ama-assn.org/ama/pub/about-ama/awards/ama-physicians-recognition-award.page. The AMA has determined that physicians not licensed in the US who participate in this CME activity are eligible for **AMA PRA Category 1 Credits™**. Through agreements that the AMA has made with agencies in some countries, AMA PRA credit may be acceptable as evidence of participation in CME activities. If you are not licensed in the US, please complete the questions online, print the AMA PRA CME credit certificate and present it to your national medical association for review.

## Post-Test Questions

### Article Title: Trends in Cigarette Smoking Rates and Quit Attempts Among Adults With and Without Diagnosed Diabetes, United States, 2001–2010

**CME Questions**

1. You are seeing a 50-year-old woman recently diagnosed with type 2 diabetes mellitus. She has smoked a half pack of cigarettes daily for the past 25 years. As you counsel this patient regarding smoking cessation, what should you consider regarding trends in cigarette smoking described in the current study by Fan and colleagues?
  - The prevalence of cigarette smoking was static between 2001 and 2010 among adults with diabetes
  - The prevalence of cigarette smoking was static between 2001 and 2010 among adults without diabetes
  - The prevalence of cigarette smoking was lower for adults with diabetes compared with adults without diabetes
  - The prevalence of cigarette smoking increased only for adults with diabetes between 2001 and 2010
2. Which of the following demographic factors was associated with a decline in smoking prevalence among persons with diabetes in the current study?
  - African American race
  - Hispanic ethnicity
  - Age 45 to 64 years
  - Educational attainment less than high school
3. The patient wants to quit smoking but feels challenged in doing so. What should you consider regarding quit attempts as demonstrated in the current study?
  - Less than one-quarter of smokers overall attempted to quit on an annual basis
  - There was no change in the overall prevalence of quit attempts
  - Rates of quit attempts increased only among persons with diabetes
  - Rates of quit attempts increased among persons with and without diabetes
4. Which of the following factors was associated with an increasing rate of quit attempts among adults with diabetes during the study period?
  - African American race
  - Non-Hispanic white race
  - Hispanic ethnicity
  - Being married or previously married

**Evaluation**

## References

[1] Haire-Joshu D , Glasgow RE , Tibbs TL . Smoking and diabetes. Diabetes Care 1999;22(11):1887–98. 10.2337/diacare.22.11.1887 10546025

[2] Tonstad S . Cigarette smoking, smoking cessation, and diabetes. Diabetes Res Clin Pract 2009;85(1):4–13. 10.1016/j.diabres.2009.04.013 19427049

[3] 2010 Surgeon General’s report — how tobacco smoke causes disease: the biology and behavioral basis for smoking-attributable disease. 2010 http://www.cdc.gov/tobacco/data_statistics/sgr/2010/index.htm. Accessed September 6, 2011. 21452462

[4] Ford ES , Mokdad AH , Gregg EW . Trends in cigarette smoking among US adults with diabetes: findings from the Behavioral Risk Factor Surveillance System. Prev Med 2004;39(6):1238–42. 10.1016/j.ypmed.2004.04.039 15539062

[5] Malarcher AM , Ford ES , Nelson DE , Chrismon JH , Mowery P , Merritt RK Trends in cigarette smoking and physicians’ advice to quit smoking among people with diabetes in the U.S. Diabetes Care 1995;18(5):694–7. 10.2337/diacare.18.5.694 8586010

[6] Centers for Disease Control and Prevention. Trends in current cigarette smoking among high school students and adults, United States, 1965–2011. http://www.cdc.gov/tobacco/data_statistics/tables/trends/cig_smoking/index.htm. Accessed June 21, 2013.

[7] Fiore MC , Jaén CR , Baker TB , Bailey WC , Benowitz NL , Curry SJ , Treating tobacco use and dependence: 2008 update — clinical practice guideline. Rockville (MD): US Department of Health and Human Services, Public Health Service; 2008.

[8] American Diabetes Association. Standards of medical care in diabetes — 2011. Diabetes Care 2011;34(Suppl 1):S11–61. 10.2337/dc11-S011 21193625 PMC3006050

[9] Centers for Disease Control and Prevention. Behavioral Risk Factor Surveillance System operational and user’s guide. Atlanta (GA): US Department of Health and Human Services. ftp://ftp.cdc.gov/pub/Data/Brfss/userguide.pdf. Accessed January 9, 2012.

[10] Mokdad AH , Stroup DF , Giles WH , Behavioral Risk Factor Surveillance Team. Public health surveillance for behavioral risk factors in a changing environment. Recommendations from the Behavioral Risk Factor Surveillance Team. MMWR Recomm Rep 2003;52(RR-9):1–12. 12817947

[11] BRFSS annual survey data. http://www.cdc.gov/brfss/. Accessed June 21, 2013.

[12] Centers for Disease Control and Prevention. National diabetes fact sheet, 2011: national estimates and general information on diabetes and prediabetes in the United States, 2011. Atlanta (GA): US Department of Health and Human Services, Centers for Disease Control and Prevention. http://www.cdc.gov/diabetes/pubs/factsheet11.htm. Accessed June 21, 2013.

[13] Ford ES , Newman J . Smoking and diabetes mellitus. Findings from 1988 Behavioral Risk Factor Surveillance System. Diabetes Care 1991;14(10):871–4. 10.2337/diacare.14.10.871 1773684

[14] McBride CM , Puleo E , Pollak KI , Clipp EC , Woolford S , Emmons KM . Understanding the role of cancer worry in creating a “teachable moment” for multiple risk factor reduction. Soc Sci Med 2008;66(3):790–800. 10.1016/j.socscimed.2007.10.014 18037204 PMC3417291

[15] Young RP , Hopkins RJ , Smith M , Hogarth DK . Smoking cessation: the potential role of risk assessment tools as motivational triggers. Postgrad Med J 2010;86(1011):26–33. 10.1136/pgmj.2009.084947 20065338

[16] Croog SH , Richards NP . Health beliefs and smoking patterns in heart patients and their wives: a longitudinal study. Am J Public Health 1977;67(10):921–30. 10.2105/AJPH.67.10.921 911003 PMC1653725

[17] McWhorter WP , Boyd GM , Mattson ME . Predictors of quitting smoking: the NHANES I followup experience. J Clin Epidemiol 1990;43(12):1399–405. 10.1016/0895-4356(90)90108-2 2254778

[18] Salive ME , Cornoni-Huntley J , LaCroix AZ , Ostfeld AM , Wallace RB , Hennekens CH . Predictors of smoking cessation and relapse in older adults. Am J Public Health 1992;82(9):1268–71. Erratum in: Am J Public Health 1992;82(11):1489. 10.2105/AJPH.82.9.1268 1503170 PMC1694340

[19] Twardella D , Loew M , Rothenbacher D , Stegmaier C , Ziegler H , Brenner H . The diagnosis of a smoking-related disease is a prominent trigger for smoking cessation in a retrospective cohort study. J Clin Epidemiol 2006;59(1):82–9. 10.1016/j.jclinepi.2005.05.003 16360565

[20] Wilkes S , Evans A . A cross-sectional study comparing the motivation for smoking cessation in apparently healthy patients who smoke to those who smoke and have ischaemic heart disease, hypertension or diabetes. Fam Pract 1999;16(6):608–10. 10.1093/fampra/16.6.608 10625137

[21] Solberg LI , Desai JR , O’Connor PJ , Bishop DB , Devlin HM . Diabetic patients who smoke: are they different? Ann Fam Med 2004;2(1):26–32. 10.1370/afm.36 15053280 PMC1466617

[22] Haire-Joshu D , Heady S , Thomas L , Schechtman K , Fisher EB Jr . Depressive symptomatology and smoking among persons with diabetes. Res Nurs Health 1994;17(4):273–82. 10.1002/nur.4770170406 8036275

[23] Willi C , Bodenmann P , Ghali WA , Faris PD , Cornuz J . Active smoking and the risk of type 2 diabetes: a systematic review and meta-analysis. JAMA 2007;298(22):2654–64. 10.1001/jama.298.22.2654 18073361

[24] Canga N , De Irala J , Vara E , Duaso MJ , Ferrer A , Martínez-Gonzalez MA . Intervention study for smoking cessation in diabetic patients: a randomized controlled trial in both clinical and primary care settings. Diabetes Care 2000;23(10):1455–60. 10.2337/diacare.23.10.1455 11023136

[25] Hokanson JM , Anderson RL , Hennrikus DJ , Lando HA , Kendall DM . Integrated tobacco cessation counseling in a diabetes self-management training program: a randomized trial of diabetes and reduction of tobacco. Diabetes Educ 2006;32(4):562–70. 10.1177/0145721706289914 16873594

[26] Millett C , Gray J , Saxena S , Netuveli G , Majeed A . Impact of a pay-for-performance incentive on support for smoking cessation and on smoking prevalence among people with diabetes. CMAJ 2007;176(12):1705–10. 10.1503/cmaj.061556 17548383 PMC1877840

[27] Persson LG , Hjalmarson A . Smoking cessation in patients with diabetes mellitus: results from a controlled study of an intervention programme in primary healthcare in Sweden. Scand J Prim Health Care 2006;24(2):75–80. 10.1080/02813430500439395 16690554

[28] Hu SS , Balluz L , Battaglia MP , Frankel MR . Improving public health surveillance using a dual-frame survey of landline and cell phone numbers. Am J Epidemiol 2011;173(6):703–11. 10.1093/aje/kwq442 21343246

[29] Centers for Disease Control and Prevention. Cigarette smoking among adults — United States, 2007. MMWR Morb Mortal Wkly Rep 2008;57(45):1221–6. Erratum in MMWR Morb Mortal Wkly Rep 2008;57(47):1281. 19008790

[30] Fiore MC , Jaén CR . A clinical blueprint to accelerate the elimination of tobacco use. JAMA 2008;299(17):2083–5. 10.1001/jama.299.17.2083 18460668

